# Telephone counseling and quitline service: An opportunity for tobacco use cessation during the COVID-19 pandemic

**DOI:** 10.5339/qmj.2021.25

**Published:** 2021-08-10

**Authors:** Ahmad AlMulla, Silva P. Kouyoumjian

**Affiliations:** , Tobacco Control Center, WHO Collaborative Center, Department of Medicine, Hamad Medical Corporation, Doha, Qatar| Hamad Medical Corporation

Effective tobacco cessation interventions, such as brief advice at the primary care level, national toll-free tobacco quitlines, cost-covered nicotine replacement therapies, and others in line with Article 14 of the World Health Organization (WHO) Framework Convention on Tobacco Control (FCTC), are vital in reducing global morbidity and mortality from tobacco use.^1,2^

Currently, only a third of countries globally have a national toll-free quitline.^1^ Over the past two years, 10 countries introduced a quitline. However, Saudi Arabia is the country from the Eastern Mediterranean region (EMR) that established a quitline, knowing that it is inexpensive to operate, particularly when using existing call centers because the infrastructure setup represents a major cost.^3^ Telephone counseling and quitlines have been recent innovative alternatives to face-to-face smoking cessation counseling, offering accessibility, convenience, and privacy to smokers.

According to WHO reports, Egypt, Iran, Kuwait, Saudi Arabia, and United Arab Emirates have a national toll-free quitlines in place.^1^ Recently, we have assessed the current tobacco quitline situation in the EMR in collaboration with the WHO Eastern Mediterranean Regional Office through an online survey conducted from June 23 to September 9, 2019. To prepare the web-based online version of the survey, Media and Corporate Communications teams at HMC provided the IT support.

The participants were the representatives of the Tobacco Control Focal Points within the Ministries of Health in 22 countries of the EMR. The EMR comprised 22 high-, middle-, and low-income countries, namely, Afghanistan, Bahrain, Djibouti, Egypt, Iran, Iraq, Jordan, Kuwait, Lebanon, Libya, Morocco, Oman, Pakistan, Palestine, Qatar, Saudi Arabia, Somalia, Sudan, Syrian Arab Republic, Tunisia, United Arab Emirates, and Yemen. Out of the 22 aforementioned countries, only 16 countries responded to our survey (72.3%). According to our findings, only Egypt and Saudi Arabia have confirmed that they have a national toll-free telephone quitline. However, notably, 10 out of the 14 countries have plans for establishing a national toll-free quitline service in the future, namely, Afghanistan, Bahrain, Iran, Jordan, Kuwait, Lebanon, Pakistan, Qatar, Tunisia, and Yemen.

Currently, Qatar does not have a toll-free national quitline; however, in response to the COVID-19 pandemic, Tobacco Control Center, a WHO Collaborating Center in HMC shifted its scope of work to meet the needs of tobacco users. In the beginning of the pandemic in March 2020 and in alignment with HMC's precautionary measures to limit the spread of the virus, face-to-face in-clinic consultations were suspended. Consequently, tobacco cessation services were provided by calling the patients via teleconsultations and/or video consultations using an application called *VSee (Sunnyvale, California)*. As most of the patients were not comfortable during video calls, the services were resumed by telephone only. The patients received appropriate in-depth counseling, treatment plan, and follow-up sessions as well as psychological support for behavior modification. The center has also received a high number of calls from smokers requesting counseling/consultation appointments over the phone.

In the EMR, tobacco smoking is the third main risk factor contributing to disease burden in general.^4^ According to a previous study, the current prevalence of cigarette smoking in Qatar was reported as 36.5%,^5^ and an updated study reported the rate of tobacco use by adults in Qatar as 25.2%.^6^ The high consumption rate of tobacco smoking in the EMR is alarming.^7^ Smoking cessation and treatment services are critical components of tobacco control and should be prioritized to significantly impact morbidity and mortality caused by tobacco.^8^ Therefore, more EMR countries should consider telephone counseling or establishing national toll-free tobacco quitline services because of its potential benefits related to identifying smokers interested in quitting, providing counseling, referrals, and further support. Quitlines are cost-effective and have a wide population reach ranging between 4% and 6% of total tobacco users per year in a country.^3^ To be effective, the quitline must provide multiple sessions of proactive counseling by a trained counselor.^9^ Proactive counseling, i.e., multiple call-backs initiated by the quitline staff after an initial inquiry by the smoker, has been found to increase cessation rates by 25–50%, which equals 2–3 absolute percentage points compared to reactive counseling model that respond only to incoming calls without additional follow-up calls.^9^ Furthermore, smokers who did not use a quitline but received telephone counseling from health providers increased their chances of smoking cessation from 11% to 14%.^10^

Although the relationship between smoking and COVID-19 severity is uncertain and controversial, there is growing evidence that smokers are at a high risk of being infected and dying due to COVID-19.^11^ Considering the COVID-19 pandemic, it is crucial to assist smokers more than ever before. Most importantly, quitline's core effectiveness and success will depend upon a proactive call-back system with three or more calls,^3,9,10^ because of the considerable potential to increase the reach of more effective treatment, in contrast to simply making referrals, which is the current practice in some EMR countries. It has been observed that quitline can aid smoking cessation in any country. For EMR countries, the successful way forward depends upon governments, and it is critical to underline that their commitments are still low. Therefore, we recommend all EMR countries to establish the national quitline within their capacity, which will be reflected positively in tobacco control.

## Authors’ Contributions

AA directed the research project. SPK wrote the short communication with input from AA. All authors have read, discussed, and approved the final manuscript.

## Funding

None.

## Acknowledgments

The authors are grateful for the support provided by the World Health Organization Eastern Mediterranean Regional Office, particularly in reviewing the survey instrument and communicating with Tobacco Control Focal Points reminding them to complete the survey. They are also thankful for the IT support provided by the Media and Corporate Communications teams at Hamad Medical Corporation in Qatar.

## Competing Interests

None to declare.
